# Eight potential biomarkers for distinguishing between lung adenocarcinoma and squamous cell carcinoma

**DOI:** 10.18632/oncotarget.17606

**Published:** 2017-05-03

**Authors:** Jian Xiao, Xiaoxiao Lu, Xi Chen, Yong Zou, Aibin Liu, Wei Li, Bixiu He, Shuya He, Qiong Chen

**Affiliations:** ^1^ Department of Geriatrics, Respiratory Medicine, Xiangya Hospital of Central South University, Changsha 410008, China; ^2^ Department of Respiratory Medicine, Xiangya Hospital of Central South University, Changsha 410008, China; ^3^ Department of Geriatrics, Xiangya Hospital of Central South University, Changsha 410008, China; ^4^ Department of Geriatrics, Clinical Laboratory, Xiangya Hospital of Central South University, Changsha 410008, China; ^5^ Department of Biochemistry & Biology, University of South China, Hengyang 421001, China

**Keywords:** lung cancer, adenocarcinoma, squamous cell carcinoma, biomarker, prognosis

## Abstract

Lung adenocarcinoma (LADC) and squamous cell carcinoma (LSCC) are the most common non-small cell lung cancer histological phenotypes. Accurate diagnosis distinguishing between these two lung cancer types has clinical significance. For this study, we analyzed four Gene Expression Omnibus (GEO) datasets (GSE28571, GSE37745, GSE43580, and GSE50081). We then imported the datasets into the Gene-Cloud of Biotechnology Information online platform to identify genes differentially expressed in LADC and LSCC. We identified DSG3 (desmoglein 3), KRT5 (keratin 5), KRT6A (keratin 6A), KRT6B (keratin 6B), NKX2-1 (NK2 homeobox 1), SFTA2 (surfactant associated 2), SFTA3 (surfactant associated 3), and TMC5 (transmembrane channel-like 5) as potential biomarkers for distinguishing between LADC and LSCC. Receiver operating characteristic curve analysis suggested that KRT5 had the highest diagnostic value for discriminating between these two cancer types. Using the PrognoScan online survival analysis tool and the Kaplan-Meier Plotter, we found that high KRT6A or KRT6B levels, or low NKX2-1, SFTA3, or TMC5 levels correlated with unfavorable prognoses in LADC patients. Further studies will be needed to verify our findings in additional patient samples, and to elucidate the mechanisms of action of these potential biomarkers in non-small cell lung cancer.

## INTRODUCTION

Non-small cell lung cancer (NSCLC) accounts for more than 85% of total lung cancer cases [1], and 5-year patient survival remains low at only 15.9% [1]. The most common NSCLC histological phenotypes are lung adenocarcinoma (LADC, ∼50% of patients) and lung squamous cell carcinoma (LSCC, ∼40% of patients) [1]. LADC cells commonly exhibit abnormal gene expression patterns and large numbers of gene mutations [2], and are characterized by specific biomarkers[3–7] and prognostic factors [8–10] that can be used to guide clinical diagnosis and treatment. LSCC cells also exhibit complex genomic alterations, including numerous gene mutations and copy number alterations [11], and are associated with particular biomarkers [12–14] and prognostic factors [15–17].

Accurate diagnosis of the LADC and LSCC cancer types has important significance for lung patient clinical treatment. While biomarkers that differentiate LADC from LSCC have been reported previously [18–21], additional markers would help enhance diagnostic accuracy for these intractable malignant cancers. The present study identified differentially expressed genes (DEGs) between LADC and LSCC samples using comprehensive bioinformatics analyses. We identified eight potential biomarkers for discriminating LADC and LSCC, and assessed their prognostic values.

## RESULTS

### Study design

**Figure 1:**
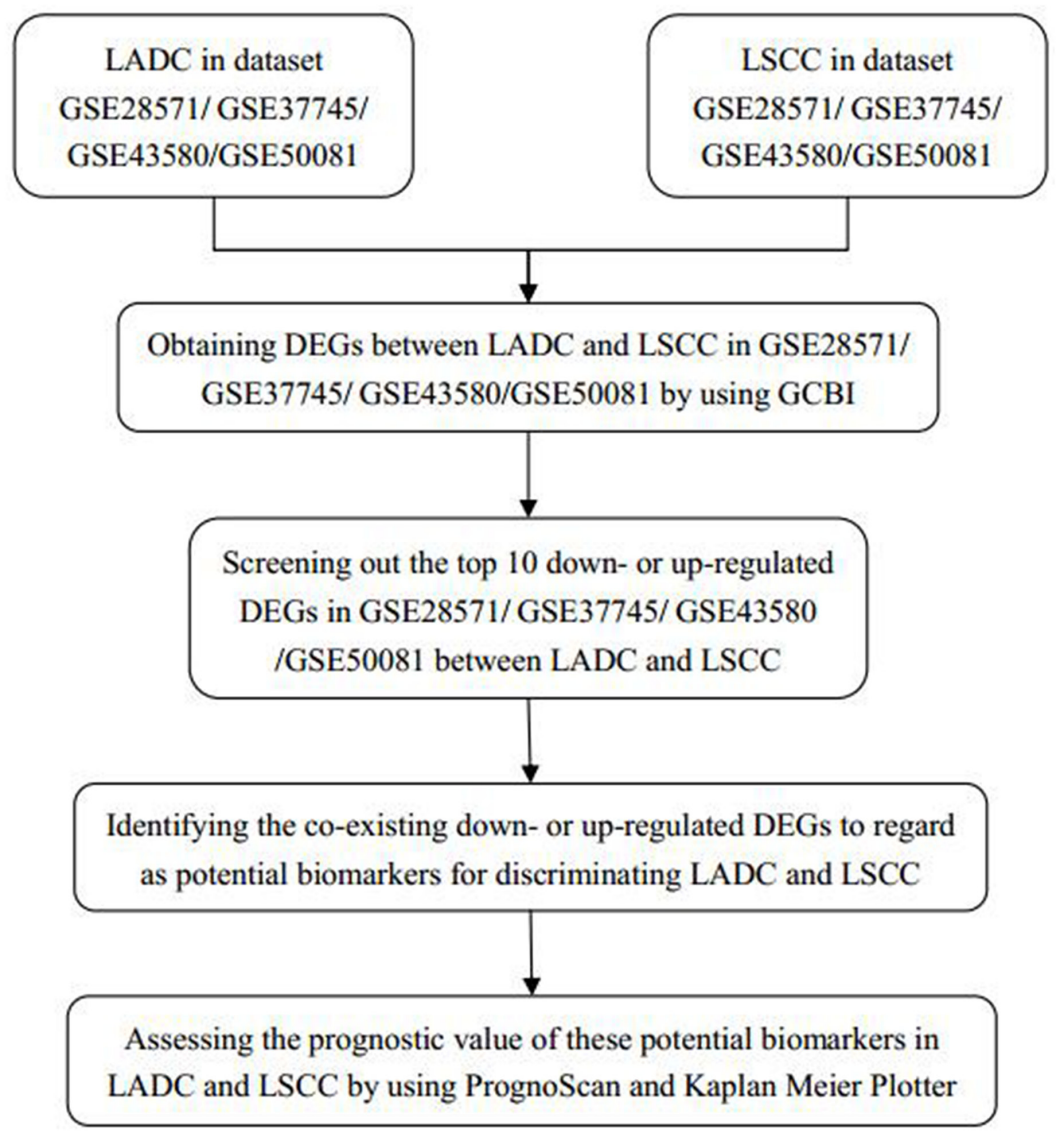
Study design diagram LADC: lung adenocarcinoma; LSCC: squamous cell carcinoma; DEGs: differentially expressed genes; GCBI: Gene-Cloud of Biotechnology Information.

We imported four Gene Expression Omnibus (GEO) datasets (GSE28571, GSE37745, GSE43580, and GSE50081) into the Gene-Cloud of Biotechnology Information (GCBI) bioinformatics analysis platform (Figure 1). We extracted LADC and LSCC gene expression information from these datasets and identified DEGs between the two cancer types. From the top 10 down- or upregulated DEGs, we identified eight as potential biomarkers for discriminating LADC and LSCC. We assessed the prognostic values of these potential biomarkers using the survival analysis tools, PrognoScan and Kaplan-Meier Plotter.

### DEGs in LADC and LSCC

**Figure 2:**
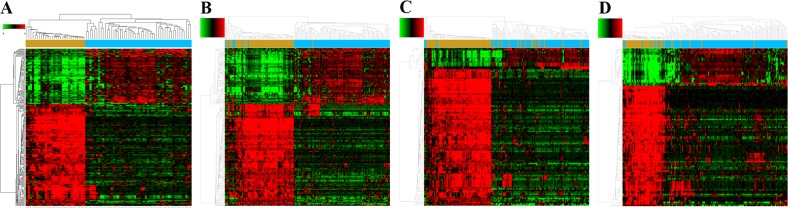
Potential DEGs between LADC and LSCC Heat maps for potential DEGs in GSE28571 (total n=243; LADC n=50; LSCC n=28) **(A)**, GSE37745 (total n=210; LADC n=106; LSCC n=66) **(B)**, GSE43580 (total n=118; LADC n=77; LSCC n=73) **(C)**, and GSE50081 (total n=101; LADC n=128; LSCC n=43) **(D).**

Using GCBI, we identified 243, 210, 118, and 101 potential DEGs from GSE28571, GSE37745, GSE43580, and GSE50081, respectively (Figure 2, Supplementary Table 1–4). Removal of duplicate genes and expression values lacking specific gene symbols left 176 DEGs from GSE28571 (Supplementary Table 5), 153 from GSE37745 (Supplementary Table 6), 81 from GSE43580 (Supplementary Table 7) and 71 from GSE50081 (Supplementary Table 8).

### Potential biomarkers for distinguishing between LADC and LSCC

**Figure 3:**
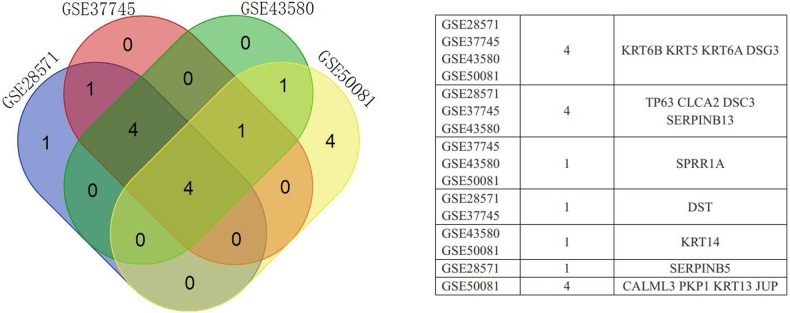
Venn diagram showing downregulated DEGs common to all four GEO datasets

**Figure 4:**
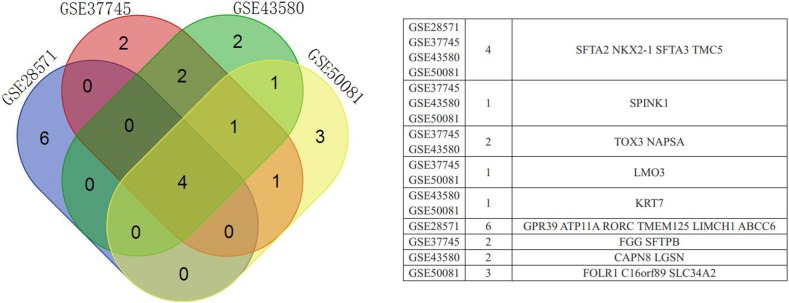
Venn diagram showing upregulated DEGs common to all four GEO datasets

Based on expression fold changes between LADC and LSCC, we selected the top 10 downregulated and upregulated DEGs from GSE28571 (Table 1), GSE37745 (Table 2), GSE43580 (Table 3), and GSE50081 (Table 4). We identified four downregulated DEGs (desmoglein 3, DSG3; keratin 5, KRT5; keratin 6A, KRT6A; keratin 6B, KRT6B) (Figure 3) and four upregulated DEGs (NK2 homeobox 1, NKX2-1; surfactant associated 2, SFTA2; surfactant associated 3, SFTA3; transmembrane channel-like 5, TMC5) (Figure 4) that were present in all four datasets. We achieved similar results via an integrated analysis based on all four datasets together (Supplementary Table 9–10). We assessed these eight genes as potential biomarkers for discriminating LADC and LSCC.

**Table 1:** Top 10 down- or upregulated DEGs between LADC and LSCC in lung cancer dataset, GSE28571

Probe set ID	Gene symbol	Gene description	Gene feature	Fold change
209125_at	KRT6A	keratin 6A	downregulation	−176.148978
206165_s_at	CLCA2	chloride channel accessory 2	downregulation	−90.443266
235075_at	DSG3	desmoglein 3	downregulation	−88.129812
201820_at	KRT5	keratin 5	downregulation	−82.362516
217272_s_at	SERPINB13	serpin peptidase inhibitor, clade B (ovalbumin), member 13	downregulation	−64.457025
213680_at	KRT6B	keratin 6B	downregulation	−52.540652
204455_at	DST	dystonin	downregulation	−46.258579
209863_s_at	TP63	tumor protein p63	downregulation	−45.820729
206032_at	DSC3	desmocollin 3	downregulation	−43.549951
204855_at	SERPINB5	serpin peptidase inhibitor, clade B (ovalbumin), member 5	downregulation	−39.535047
244056_at	SFTA2	surfactant associated 2	upregulation	31.032507
228979_at	SFTA3	surfactant associated 3	upregulation	27.153369
211024_s_at	NKX2-1	NK2 homeobox 1	upregulation	15.422392
219580_s_at	TMC5	transmembrane channel-like 5	upregulation	11.725501
229105_at	GPR39	G protein-coupled receptor 39	upregulation	6.443132
214033_at	ABCC6	ATP-binding cassette, sub-family C (CFTR/MRP), member 6	upregulation	6.288185
212328_at	LIMCH1	LIM and calponin homology domains 1	upregulation	6.28786
225822_at	TMEM125	transmembrane protein 125	upregulation	5.919894
230875_s_at	ATP11A	ATPase, class VI, type 11A	upregulation	5.787312
228806_at	RORC	RAR-related orphan receptor C	upregulation	5.335111

**Table 2:** Top 10 down- or upregulated DEGS between LADC and LSCC in lung cancer dataset, GSE37745

Probe set ID	Gene symbol	Gene description	Gene feature	Fold change
209125_at	KRT6A	keratin 6A	downregulation	−140.927
235075_at	DSG3	desmoglein 3	downregulation	−86.646
206165_s_at	CLCA2	chloride channel accessory 2	downregulation	−84.9649
201820_at	KRT5	keratin 5	downregulation	−62.2157
213680_at	KRT6B	keratin 6B	downregulation	−53.2072
206032_at	DSC3	desmocollin 3	downregulation	−47.29
209863_s_at	TP63	tumor protein p63	downregulation	−44.3825
204455_at	DST	dystonin	downregulation	−38.1615
213796_at	SPRR1A	small proline-rich protein 1A	downregulation	−36.8294
217272_s_at	SERPINB13	serpin peptidase inhibitor, clade B (ovalbumin), member 13	downregulation	−36.3898
228979_at	SFTA3	surfactant associated 3	upregulation	33.59706
244056_at	SFTA2	surfactant associated 2	upregulation	27.97213
216623_x_at	TOX3	TOX high mobility group box family member 3	upregulation	21.41014
206239_s_at	SPINK1	serine peptidase inhibitor, Kazal type 1	upregulation	17.47105
211024_s_at	NKX2-1	NK2 homeobox 1	upregulation	16.6846
223806_s_at	NAPSA	napsin A aspartic peptidase	upregulation	14.23227
37004_at	SFTPB	surfactant protein B	upregulation	12.19793
240304_s_at	TMC5	transmembrane channel-like 5	upregulation	11.27782
204424_s_at	LMO3	LIM domain only 3 (rhombotin-like 2)	upregulation	10.23422
219612_s_at	FGG	fibrinogen gamma chain	upregulation	9.826917

**Table 3:** Top 10 down- or upregulated DEGs between LADC and LSCC in lung cancer dataset, GSE43580

Probe set ID	Gene symbol	Gene description	Gene feature	Fold change
209125_at	KRT6A	keratin 6A	downregulation	−53.2466
235075_at	DSG3	desmoglein 3	downregulation	−45.44
206165_s_at	CLCA2	chloride channel accessory 2	downregulation	−38.0985
209863_s_at	TP63	tumor protein p63	downregulation	−28.6096
213796_at	SPRR1A	small proline-rich protein 1A	downregulation	−27.828
201820_at	KRT5	keratin 5	downregulation	−26.5195
206032_at	DSC3	desmocollin 3	downregulation	−25.687
213680_at	KRT6B	keratin 6B	downregulation	−25.5837
217272_s_at	SERPINB13	serpin peptidase inhibitor, clade B (ovalbumin), member 13	downregulation	−22.7939
209351_at	KRT14	keratin 14	downregulation	−21.4751
216623_x_at	TOX3	TOX high mobility group box family member 3	upregulation	12.48837
228979_at	SFTA3	surfactant associated 3	upregulation	9.698342
244056_at	SFTA2	surfactant associated 2	upregulation	9.34222
220393_at	LGSN	lengsin, lens protein with glutamine synthetase domain	upregulation	7.272057
223806_s_at	NAPSA	napsin A aspartic peptidase	upregulation	6.387242
211024_s_at	NKX2-1	NK2 homeobox 1	upregulation	6.235382
240304_s_at	TMC5	transmembrane channel-like 5	upregulation	5.886752
229030_at	CAPN8	calpain 8	upregulation	5.558286
209016_s_at	KRT7	keratin 7	upregulation	5.197863
206239_s_at	SPINK1	serine peptidase inhibitor, Kazal type 1	upregulation	5.028636

**Table 4:** Top 10 down- or upregulated DEGs between LADC and LSCC in lung cancer dataset, GSE50081

Probe set ID	Gene symbol	Gene description	Gene feature	Fold change
209125_at	KRT6A	keratin 6A	downregulation	−57.006103
213680_at	KRT6B	keratin 6B	downregulation	−39.001783
201820_at	KRT5	keratin 5	downregulation	−37.082683
207935_s_at	KRT13	keratin 13	downregulation	−23.955773
210020_x_at	CALML3	calmodulin-like 3	downregulation	−22.527441
235075_at	DSG3	desmoglein 3	downregulation	−21.167905
213796_at	SPRR1A	small proline-rich protein 1A	downregulation	−20.461997
221854_at	PKP1	plakophilin 1 (ectodermal dysplasia/skin fragility syndrome)	downregulation	−18.214428
205157_s_at	JUP	junction plakoglobin	downregulation	−17.594235
209351_at	KRT14	keratin 14	downregulation	−16.96603
228979_at	SFTA3	surfactant associated 3	upregulation	13.36924
244056_at	SFTA2	surfactant associated 2	upregulation	13.198138
211024_s_at	NKX2-1	NK2 homeobox 1	upregulation	11.03073
240304_s_at	TMC5	transmembrane channel-like 5	upregulation	8.335526
206239_s_at	SPINK1	serine peptidase inhibitor, Kazal type 1	upregulation	7.171856
209016_s_at	KRT7	keratin 7	upregulation	6.780702
204124_at	SLC34A2	solute carrier family 34 (sodium phosphate), member 2	upregulation	6.362828
204437_s_at	FOLR1	folate receptor 1 (adult)	upregulation	6.138674
229177_at	C16orf89	chromosome 16 open reading frame 89	upregulation	6.035951
204424_s_at	LMO3	LIM domain only 3 (rhombotin-like 2)	upregulation	5.987309

**Figure 5:**
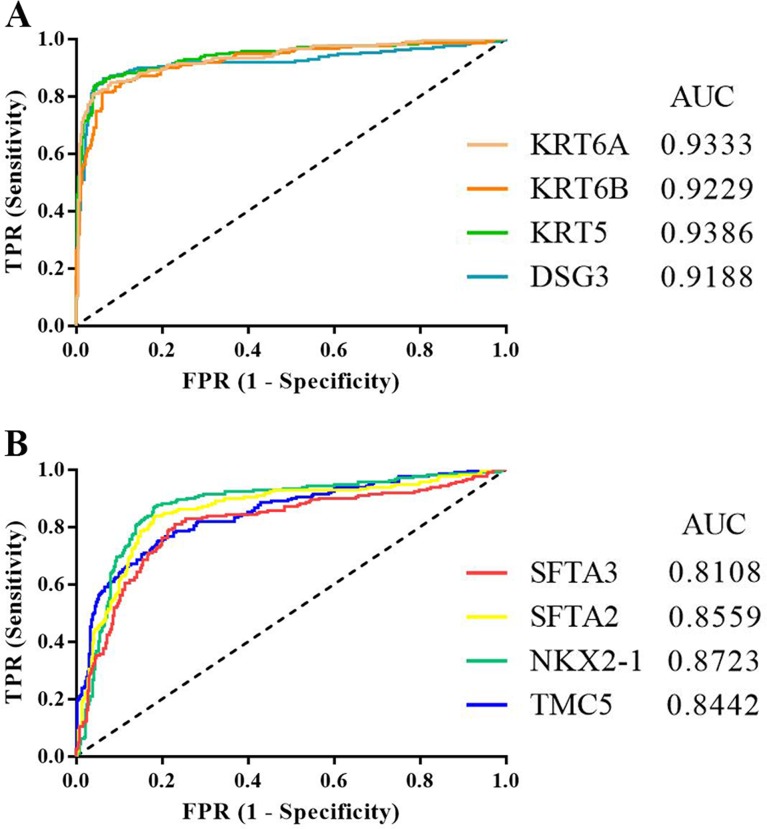
ROC curves for downregulated **(A)** and upregulated DEGs **(B)** in distinguishing between LADC and LSCC. TPR: true positive rate; FPR: false positive rate; AUC: area under the curve.

Receiver operating characteristic (ROC) curve analysis was used to evaluate the diagnostic values of DSG3, KRT5, KRT6A, KRT6B, NKX2-1, SFTA2, SFTA3, and TMC5. The four downregulated DEGs had similar areas under the curve (AUC): 0.9188 for DSG3, 0.9386 for KRT5, 0.9333 for KRT6A, and 0.9229 for KRT6B (Figure 5A). The four upregulated DEGs also had similar AUCs: 0.8723 for NKX2-1, 0.8559 for SFTA2, 0.8108 for SFTA3, and 0.8442 for TMC5 (Figure 5B). AUC results showed that KRT5 had the highest diagnostic value for discriminating LADC and LSCC.

### PrognoScan identified potential prognostic factors for LADC and LSCC patients

We assessed the prognostic values of the eight potential biomarkers using the bioinformatics analysis platform, PrognoScan. *P*<0.05 was considered significant in Cox regression analyses. We found that high DSG3, KRT6A, or KRT6B levels (Table 5), or low NKX2-1, SFTA3, or TMC5 levels (Table 6), were associated with unfavorable prognosis in LADC patients. However, only low NKX2-1 expression was associated with unfavorable prognosis in LSCC patients (Table 6). We speculated that DSG3, KRT6A, KRT6B, NKX2-1, SFTA3, and TMC5 might be LADC patient prognostic factors, and NKX2-1 might be an LSCC patient prognostic factor. Because each lung cancer microarray dataset in PrognoScan contained limited cases (Table 5–6), we verified these findings using Kaplan-Meier Plotter.

**Table 5:** DSG3, KRT5, KRT6A, and KRT6B prognostic values in LADC and LSCC as assessed by PrognoScan

Gene symbol	LADC				LSCC			
	Dataset	Case	HR (95% CIs)	*P*-value	Dataset	Case	HR (95% CIs)	*P*-value
**DSG3**	MICHIGAN-LC	86	2.54 (1.22-5.32)	0.013244	-	-	-	>0.05
KRT5	-	-	-	>0.05	-	-	-	>0.05
**KRT6A**	jacob-00182-HLM	79	1.24 (1.06–1.45)	0.006974	-	-	-	>0.05
	jacob-00182-MSK	104	1.28 (1.06–1.53)	0.008562				
	GSE31210	204	1.39 (1.18–1.63)	0.000083				
**KRT6B**	jacob-00182-MSK	104	1.26 (1.07–1.47)	0.005120	-	-	-	>0.05
	GSE31210	204	1.47 (1.23–1.75)	0.000017				

**Table 6:** NKX2-1, SFTA2, SFTA3, and TMC5 prognostic values in LADC and LSCC as assessed by PrognoScan

Gene symbol	LADC				LSCC			
	Dataset	Case	HR (95% CIs)	*P*-value	Dataset	Case	HR (95% CIs)	*P*-value
**NKX2-1**	jacob-00182-CANDF	82	0.78 (0.64–0.96)	0.020132	GSE17710	56	0.71 (0.52-0.97)	0.029764
	jacob-00182-HLM	79	0.78 (0.63–0.97)	0.027745				
	MICHIGAN-LC	86	0.56 (0.36–0.87)	0.009902				
	GSE31210	204	0.62 (0.43–0.88)	0.008218				
	jacob-00182-UM	178	0.81 (0.68–0.97)	0.021112				
SFTA2	-	-	-	>0.05	-	-	-	-
**SFTA3**	GSE13213	117	0.89 (0.79–1.00)	0.048445	-	-	-	-
	GSE31210	204	0.62 (0.46–0.85)	0.003019				
**TMC5**	jacob-00182-HLM	79	0.45 (0.24–0.84)	0.012012	-	-	-	>0.05
	GSE31210	204	0.30 (0.13–0.68)	0.004014				

### Kaplan-meier plotter verified five LADC prognostic factors

**Figure 6:**
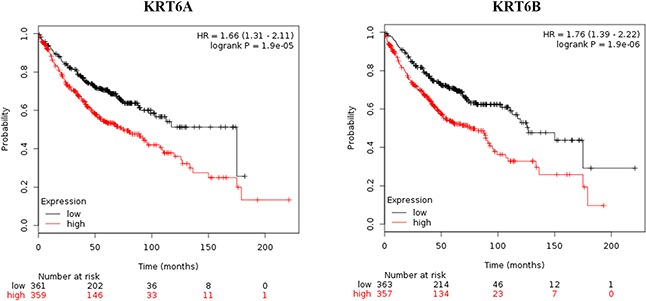
Kaplan-Meier survival curves for KRT6A and KRT6B expression in LADC patients

**Figure 7:**
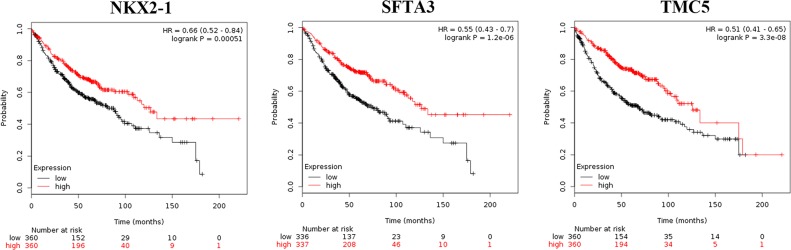
Kaplan-Meier survival curves for NKX2-1, SFTA3, and TMC5 expression in LADC patients

Using Kaplan-Meier Plotter, we verified that high KRT6A (Hazard ratio, HR=1.66; 95% confidence intervals, 95% CIs: 1.31–2.11; *P*=1.90E-05) or KRT6B (HR=1.76; 95% CIs: 1.39–2.22; *P*=1.90E-06) (Figure 6, Table 7), or low NKX2-1 (HR=0.66; 95% CIs: 0.52–0.84; *P*=0.00051), SFTA3 (HR=0.55; 95% CIs: 0.43–0.70; *P*=1.20E-06), or TMC5 (HR=0.51; 95% CIs: 0.41–0.65; *P*=3.30E-08) (Figure 7, Table 7) levels correlated with unfavorable prognosis in LADC patients. However, no DEGs correlated with LSCC patient prognosis (Table 7). Unlike the scattered results obtained by PrognoScan, Kaplan-Meier Plotter gained the meta-analysis results and we therefore draw our conclusions based on the Kaplan-Meier Plotter findings.

**Table 7:** Verification of potential prognostic indicators via Kaplan-Meier Plotter

Gene symbol	LADC			LSCC		
	Case	HR (95% CIs)	*P*-value	Case	HR (95% CIs)	*P*-value
DSG3	673	1.09 (0.86-1.39)	0.48	271	0.86 (0.63–1.18)	0.35
**KRT6A**	720	**1.66 (1.31–2.11)**	**1.90E-05**	524	0.99 (0.78–1.25)	0.92
**KRT6B**	720	**1.76 (1.39–2.22)**	**1.90E-06**	524	0.94 (0.75–1.20)	0.63
**NKX2-1**	720	**0.66 (0.52–0.84)**	**0.00051**	524	0.82 (0.65–1.04)	0.11
**SFTA3**	673	**0.55 (0.43–0.70)**	**1.20E-06**	271	0.82 (0.60–1.11)	0.20
**TMC5**	720	**0.51 (0.41–0.65)**	**3.30E-08**	524	1.02 (0.8–1.29)	0.88

## DISCUSSION

In this study, we imported four GEO datasets into the GCBI comprehensive analysis platform to extract LADC and LSCC gene expression data. We identified DEGs between LADC and LSCC samples through differential expression analysis in GCBI, and found that DSG3, KRT5, KRT6A, KRT6B, NKX2-1, SFTA2, SFTA3, and TMC5 were potential biomarkers for distinguishing the two cancer types. According to ROC analyses, KRT5 had the highest diagnostic value for discriminating LADC and LSCC. Finally, using the survival analysis platforms, PrognoScan and Kaplan-Meier Plotter, we found that high KRT6A or KRT6B, or low NKX2-1, SFTA3, or TMC5 levels correlated with unfavorable prognoses in LADC patients.

Previous studies reported that DSG3 [18, 21, 22], KRT5 [23], KRT6A [24], and KRT6B [24] levels were higher in LSCC than in LADC, and that NKX2-1 [25–27], SFTA3 [21], and TMC5 [21] levels were higher in LADC than in LSCC, suggesting that these genes were biomarkers for differentiating between LSCC and LADC. In agreement with this, our results showed that DSG3, KRT5, KRT6A, and KRT6B were downregulated in LADC compared to LSCC, and that NKX2-1, SFTA3, and TMC5 were upregulated in LADC compared to LSCC. Our study also identified SFTA2 as a novel biomarker upregulated in LADC.

The potential biomarker, NKX2-1, binds DNA damage-binding protein 1 (DDB1) and degrades check-point kinase 1 (CHK1) to facilitate lung adenocarcinoma progression [28]. Through modulating IKKβ/NF-κB pathway activation, NKX2-1 also modulates lung adenocarcinoma by directly regulating p53 transcription [29]. However, the molecular mechanisms by which DSG3, KRT5, KRT6A, KRT6B, SFTA2, SFTA3, and TMC5 regulate NSCLC development remain unclear. DSG3 promotes epidermoid carcinoma progression by regulating activation of protein kinase C-dependent Ezrin and activator protein 1 [30]. KRT5 combines with transforming growth factor beta receptor 3 (TGFBR3) and transcription factor JunD to promote breast cancer cell growth [31]. KRT6B interacts with notch1 to promote renal carcinoma development [32]. Studies to elucidate the mechanisms of action of these biomarkers in NSCLC development and progression are warranted.

Lu C, *et al.* [33] and Tian [34] also extracted gene expression data from GEO profiles to identify DEGs between LADC and LSCC. Based on the GSE6044 and GSE50081 datasets, these groups identified 19 and 33 DEGs, respectively, that might discriminate between LADC and LSCC. However, these genes were not identified based on expression fold changes between LADC and LSCC. Fold change is important for detecting DEGs [35–37] and guiding further research [38, 39], and our eight potential biomarkers for differentiating between LADC and LSCC were identified based on this measurement type in the GSE28571, GSE37745, GSE43580, and GSE50081 datasets. Consequently, the biomarkers reported here differ from those identified in previous studies [33, 34]. This indicates that different gene expression dataset screening methods may produce different results and the differences of molecule expression between LADC and LSCC may be far more complicated than we thought.

Previous studies have identified prognostic biomarkers in patients with LADC [10, 40–44] or LSCC [45–49]. While we did not identify any LSCC prognostic indictors, we found that high KRT6A or KRT6B levels, or low NKX2-1, SFTA3, or TMC5 levels correlated with an unfavorable prognosis in LADC patients. Of these prognostic factors, only NKX2-1, thought to be a tumor suppressor [50], was previously associated with LADC prognosis [26, 51]. The prognostic values of KRT6A, KRT6B, SFTA3, and TMC5 in LADC are reported here for the first time. Both KRT6A and KRT6B are type II cytokeratins and keratin 6 isoforms [52, 53]. KRT6A and KRT6B are associated with pachyonychia congenita [54, 55], as well as renal carcinoma [32] and breast cancer [56] progression. SFTA3 is an immunoregulatory protein that protects lung tissue during inflammation and is likely a lung surfactant protein family member [57]. SFTA3 is also downregulated in anaplastic thyroid carcinoma compared with normal thyroid tissue [58]. TMC5 is a transmembrane protein with at least eight membrane-spanning domains that belongs to a novel group of transporters, ion channels, or modifiers of such [59]. TMC5 is upregulated in chromophobe renal cell carcinoma [60] and intrahepatic cholangiocarcinoma [61].

In conclusion, we identified DSG3, KRT5, KRT6A, KRT6B, NKX2-1, SFTA2, SFTA3, and TMC5 as potential biomarkers for distinguishing between LADC and LSCC. Additionally, high KRT6A or KRT6B levels, or low NKX2-1, SFTA3, or TMC5 levels correlated with unfavorable LDAC patient prognosis. Further studies are required to verify our findings in additional patient samples, and to elucidate the mechanisms of action of these potential biomarkers in NSCLC.

## MATERIALS AND METHODS

### Gene expression omnibus datasets

The Gene Expression Omnibus (GEO) (https://www.ncbi.nlm.nih.gov/gds) is a public repository at the National Center of Biotechnology Information for storing high throughput gene expression datasets. We screened potential GEO datasets according to the following inclusion criteria: 1) *Homo sapiens* NSCLC specimens classified as LADC or LSCC; 2) expression profiling by array; 3) performed on the GPL570 platform ([HG-U133_Plus_2] Affymetrix Human Genome U133 Plus 2.0 Array); and 4) ≥100 samples. Datasets with specimens from other organisms, expression profiling by RT-PCR (or genome variation profiling by SNP array/SNP genotyping by SNP array), analyses on platforms other than GPL570, or sample size <100 were excluded.

We used the search terms, “((lung cancer [Title]) AND GPL570 [Related Series]) AND *Homo sapiens* [Organism] AND (squamous cell carcinoma [Description] OR adenocarcinoma [Description]),” to identify potential datasets within GEO. Screening using the aforementioned inclusion criteria identified four datasets (GSE28571, GSE37745, GSE43580, and GSE50081) for use in analyses of DEGs between LADC and LSCC. These datasets contained 361 LADC (50 in GSE28571, 106 in GSE37745, 77 in GSE43580, and 128 in GS50081) and 210 LSCC (28 in GSE28571, 66 in GSE37745, 73 in GSE43580, and 43 in GSE50081) fresh-frozen specimens (Tables S11–S14).

### Gene-cloud of biotechnology information

Gene-Cloud of Biotechnology Information (GCBI; https://www.gcbi.com.cn/gclib/html/index), is an online comprehensive bioinformatics analysis platform that can systematically analyze GEO dataset-derived gene expression information [62]. After flagged data normalization, filtering, and quality control, we identified genes differentially expressed by >5 fold between LADC and LSCC, with the cutoff values *P*<0.05 and Q<0.05 using GCBI.

### Prognoscan

The PrognoScan (http://www.prognoscan.org/) online database provides a powerful platform for exploring therapeutic targets, tumor markers, and prognostic factors in cancer patients [63], and contains cancer microarray datasets with corresponding clinical data. PrognoScan automatically calculates HRs, 95% CIs, and Cox *P*-values according to a given gene's mRNA level (high or low).

### Kaplan-meier plotter

Kaplan-Meier Plotter (http://kmplot.com/analysis/) is an online database of published microarray datasets for four cancer types (breast, ovarian, lung, and gastric cancer), and includes clinical data and gene expression information for 2,437 lung cancer patients [64]. Kaplan-Meier Plotter is useful for assessing new biomarkers related to lung cancer patient survival.

### Receiver operating characteristic curve analyses

Receiver operating characteristic (ROC) curves were constructed to compare biomarker diagnostic values. Curves are created by plotting true positive rates (TPR, sensitivity) against false positive rates (FPR, 1-specificity). The area under the curve (AUC) is used to determine diagnostic accuracy. An AUC value close to 1.0 indicates high accuracy [65].

## SUPPLEMENTARY MATERIALS TABLES






























