# Supplementary material for: Eight potential biomarkers for distinguishing between lung adenocarcinoma and squamous cell carcinoma
Source: Oncotarget. 2017 May 3;8(42):71759–71. doi: 10.18632/oncotarget.17606 (PMC5641087; doi:10.18632/oncotarget.17606)
Supplement: Supplementary file 1 [file oncotarget-08-71759-s001.pdf]

Eight potential biomarkers for distinguishing between lung adenocarcinoma and squamous cell carcinoma

SUPPLEMENTARY TABLES

Supplementary Table 1: Potentially differentially expressed genes between lung adenocarcinoma and squamous cell carcinoma in dataset GSE28571.

See Supplementary File 1

Supplementary Table 2: Potentially differentially expressed genes between lung adenocarcinoma and squamous cell carcinoma in dataset GSE37745.

See Supplementary File 2

Supplementary Table 3: Potentially differentially expressed genes between lung adenocarcinoma and squamous cell carcinoma in dataset GSE43580.

See Supplementary File 3

Supplementary Table 4: Potentially differentially expressed genes between lung adenocarcinoma and squamous cell carcinoma in dataset GSE50081.

See Supplementary File 4

Supplementary Table 5: Differentially expressed genes (that removed the expressions without specific gene symbol and the reduplicative expressions under a same gene symbol) between lung adenocarcinoma and squamous cell carcinoma in dataset GSE28571.

See Supplementary File 5

Supplementary Table 6: Differentially expressed genes (that removed the expressions without specific gene symbol and the reduplicative expressions under a same gene symbol) between lung adenocarcinoma and squamous cell carcinoma in dataset GSE37745.

See Supplementary File 6

Supplementary Table 7: Differentially expressed genes (that removed the expressions without specific gene symbol and the reduplicative expressions under a same gene symbol) between lung adenocarcinoma and squamous cell carcinoma in dataset GSE43580.

See Supplementary File 7

Supplementary Table 8: Differentially expressed genes (that removed the expressions without specific gene symbol and the reduplicative expressions under a same gene symbol) between lung adenocarcinoma and squamous cell carcinoma in dataset GSE50081.

See Supplementary File 8

Supplementary Table 9: Potentially differentially expressed genes between lung adenocarcinoma and squamous cell carcinoma merged by four datasets (GSE28571, GSE37745, GSE43580 and GSE50081).

See Supplementary File 9

Supplementary Table 10: Differentially expressed genes (that removed the expressions without specific gene symbol and the reduplicative expressions under a same gene symbol) between lung adenocarcinoma and squamous cell carcinoma merged by four datasets (GSE28571, GSE37745, GSE43580 and GSE50081).

See Supplementary File 10

Supplementary Table 11: Main characteristics of adenocarcinoma and squamous cell carcinoma samples in lung cancer dataset GSE28571 (For more details, please visit the website of https://www.gcbi.com.cn/gclib/html/sample/R1NFMjg1NzE/1/20).

See Supplementary File 11

Supplementary Table 12: Main characteristics of adenocarcinoma and squamous cell carcinoma samples in lung cancer dataset GSE37745 (For more details, please visit the website of https://www.gcbi.com.cn/gclib/html/sample/R1NFMzc3NDU/1/20).

See Supplementary File 12

Supplementary Table 13: Main characteristics of adenocarcinoma and squamous cell carcinoma samples in lung cancer dataset GSE43580 (For more details, please visit the website of https://www.gcbi.com.cn/gclib/html/sample/R1NFNDM1ODA/1/20).

See Supplementary File 13

Supplementary Table 14: Main characteristics of adenocarcinoma and squamous cell carcinoma samples in lung cancer dataset GSE50081 (For more details, please visit the website of https://www.gcbi.com.cn/gclib/html/sample/R1NFNTAwODE/1/20).

See Supplementary File 14
